# Graphene-Based Electrochemical Sensing Platform for Rapid and Selective Ferulic Acid Quantification

**DOI:** 10.3390/ijms242316937

**Published:** 2023-11-29

**Authors:** Lidia Mǎgeruşan, Florina Pogǎcean, Maria-Loredana Soran, Stela-Maria Pruneanu

**Affiliations:** National Institute for Research and Development of Isotopic and Molecular Technologies, Donat Street, No. 67-103, 400293 Cluj-Napoca, Romania; florina.pogacean@itim-cj.ro (F.P.); loredana.soran@itim-cj.ro (M.-L.S.); stela.pruneanu@itim-cj.ro (S.-M.P.)

**Keywords:** sulphur-doped graphenes, electrochemical analysis, cyclic voltammetry, modified electrodes, ferulic acid

## Abstract

Due to the multitude of physiological functions, ferulic acid (FA) has a wide range of applications in the food, cosmetic, and pharmaceutical industries. Thus, the development of rapid, sensitive, and selective detection tools for its assay is of great interest. This study reports a new electroanalytical approach for the quantification of ferulic acid in commercial pharmaceutical samples using a sulphur-doped graphene-based electrochemical sensing platform. The few-layer graphene material (exf-SGR) was prepared by the electrochemical oxidation of graphite, at a low applied bias (5 V), in an inorganic salt mixture of Na_2_S_2_O_3_/(NH_4_)_2_SO_4_ (0.3 M each). According to the morpho-structural characterization of the material, it appears to have a high heteroatom doping degree, as proved by the presence of sulphur lines in the XRD pattern, and the C/S ratio was determined by XPS investigations to be 11.57. The electrochemical performances of a glassy carbon electrode modified with the exf-SGR toward FA detection were tested by cyclic voltammetry in both standard laboratory solutions and real sample analysis. The developed modified electrode showed a low limit of detection (30.3 nM) and excellent stability and reproducibility, proving its potential applicability as a viable solution in FA qualitative and quantitative analysis.

## 1. Introduction

Among various polyphenols, ferulic acid (FA, 3-methoxy-4-hydroxy cinnamic acid), a common bioactive element in fruits and vegetables, is the most effective antioxidant, playing an important function in cell components’ protection [1]. FA protects the primary skin tissues, including keratinocytes, fibroblasts, collagen, and elastin. It suppresses melanogenesis, promotes angiogenesis, and quickens the healing process after injury [2]. Moreover, in addition to being a free radical scavenger, ferulic acid also inhibits enzymes that accelerate the production of free radicals and increases the activity of scavenging enzymes [3]. FA is considered to be a superior antioxidant, since it is more quickly and easily absorbed by the organism and remains in the bloodstream longer than any other phenolic acid. Recent years have seen an upsurge in studies on ferulic acid’s physiological effects including its anti-inflammatory [4,5]; anti-oxidant [6,7]; anti-thrombotic [8]; antiproliferative, proapoptotic, and angiogenic [9,10,11]; antimicrobial and antibacterial [12,13]; antiarrhythmic [14,15]; anticancer (including skin, lung, breast, colon, and other types of cancer) [16,17]; and antidiabetic activities [18,19], as well as its immunostimulant properties [20,21]. 

However, FA research as a whole is still in its early stage, since up until now the maximum admitted intake dosage has no established standards, and the long-term safety is not well understood. In some reported cases, besides the pharmacological beneficial effects, some concerns and issues regarding FA cytotoxicity have been raised. FA was discovered to be harmful at a greater dosage that exceeds 50 µM [22]. Furthermore, Truzzi et al.’s [23] results showed that, at high concentrations, FA can be toxic to in vitro cell models, indicating that excessive and unregulated ingestion may have deleterious effects on the intestinal wall. 

In the given context, before FA safety and effectiveness—for any use—can be guaranteed, much more research is required. Thus, the development of sensitive analytical techniques is crucial, especially for assessing the quality of commercialized products. At present, the various chromatographic (high-performance liquid chromatography—HPLC [24]; liquid chromatography with tandem mass spectrometry—LC-MS-MS [25]; ultra-performance liquid chromatography—UPLC [26]) and spectrophotometric [27] analytical tools employed for FA detection and quantification are linked to significant drawbacks due to the complex, multistep sample preparation procedures; expensive equipment and analytical columns; necessity of large quantities of environmentally risky solvents in the mobile phase; and high maintenance costs. Because of various factors such as good selectivity, a rapid response, low cost, potential downsizing, and the simplicity of operation, the electroanalytical approach emerged as an alternate method [28] for an FA assay. Over time, different functional electrodes, such as an L-cysteine self-assembled monolayer (SAM) modified gold electrode [29]; a carbon nanofiber-based screen-printed sensor [30]; graphene oxide sheets (GOs) and a multi-walled carbon nanotubes (MWCNTs)’ nanocomposites modified glassy carbon electrode [31]; a poly(diallyldimethylammonium chloride) (PDDA)-functionalized graphene-modified gold electrode [32]; a poly-aspartic acid-modified electrode [33]; a polypyrrole-multiwalled carbon nanotubes-modified electrode [34]; a reduced—graphene oxide (ERGO)-modified glassy carbon electrode (GCE) [35]; polyaminobenzene sulfonic acid functionalized single-walled carbon nanotubes (f-SWCNTs) and an electropolymerized bromocresol purple glassy carbon electrode [36]; and carbon nanofibers–gold nanoparticles–tyrosinase-modified screen-printed electrodes (SPEs) [37]. Carbon nanotubes decorated with manganese dioxide nanoparticles-modified glassy carbon electrode [38] were developed and used to assess FA electrochemical behavior. However, the few studies reporting the development of electrochemical sensors for FA detection are governed by serious drawbacks related to the complex and cost-prohibitive synthesis procedures employed for obtaining electrode materials. Thus, the development of sensors with high sensitivity, selectivity, and reaction times relative to traditional analytical procedures remains a significant issue. Among all of its superior mechanical properties [39], due to its remarkable and unique properties (increased carrier mobility [40] and high thermal and electrical conductivity [41]), graphene has revolutionized the scientific frontiers of the modified electrodes area. Heteroatom doping has the potential to enhance graphene properties, increasing the surface area and improving the material surface wettability by increasing the number of hydrophilic sites [42], considerably increasing the capacity and energy density [43]. Previous studies demonstrated that sulphur-doped graphene has strong catalytic properties for oxidation–reduction reactions due to the high spin density resulting from sulphur atoms’ doping, since the heteroatoms induce a considerable number of defects in the graphene lattice [44]. However, to the best of our knowledge, up until now, no sulphur-doped modified electrodes have been employed for an FA electrochemical assay.

Thus, the aim of this study was to achieve FA-sensitive voltammetric detection and quantification employing a simple electrochemical sensor based on the sulphur-doped graphene glassy carbon modified electrode (exf-SGR/GCE). Compared to other scientific reports involving FA detection, the reported synthesis procedure for obtaining the heteroatom-doped graphene (exf-SGR) is simple, rapid, and economically advantageous, avoiding the usage of toxic organic solvents. Furthermore, due to its ease of use, great sensitivity, and selectivity, as well as a quick reaction time in comparison with traditional analytical procedures, the developed sensor has the potential for the quick screening of FA in actual samples, which would broaden the applicability of a graphene-based nanocomposite in the pharmaceutical analysis industry. 

## 2. Results and Discussion

### 2.1. Morpho-Structural Sulphur-Doped Graphene Characterization

**Figure 1:**
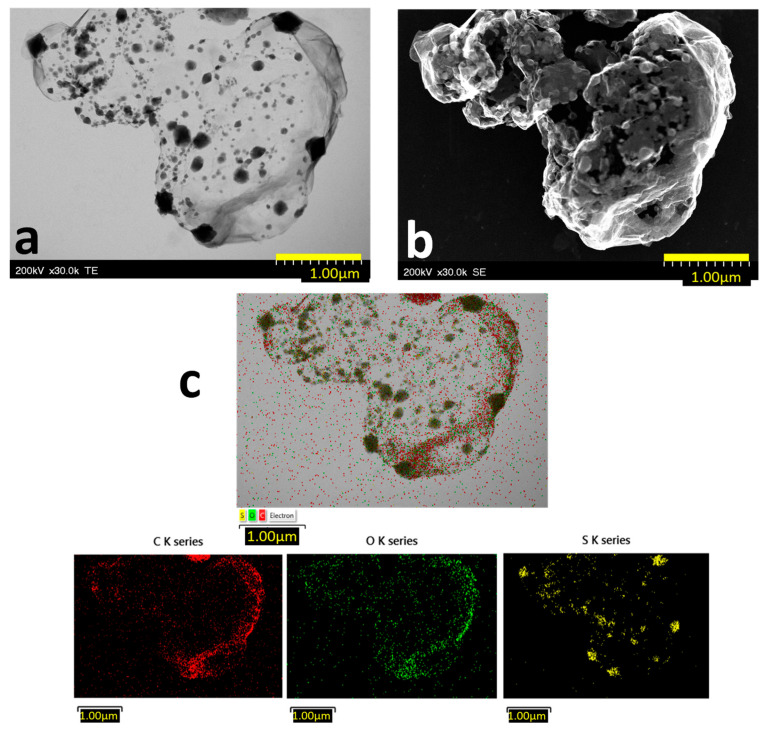
(**a**) TEM and (**b**) SEM images of exf-SGR material; (**c**) STEM-EDS measurements and elemental maps of C, O, and S atoms’ distribution (scale bar 1 µm).

TEM characterization enables the precise determination of a sulphur-doped graphene’s nanoscale structure and composition, revealing the presence of defects and other morphological features. TEM/SEM analysis shows that the morphology of the exf-SGR sample is dominated by large, micrometer-range graphene sheets with a thin, smooth–silky appearance, having embedded from point to point some spherical structures (dark points in Figure 1a and light points in Figure 1b). STEM-EDS mapping analysis (shown in Figure 1c) confirmed that these dark-colored features in the graphene-based material are due to the incorporation of sulphur in its two-dimensional structure. According to the microscopic investigation, the sulphur appeared to be uniformly distributed on the surface of graphene sheets. Moreover, the mean diameter (D) of the sulphur crystallites, which adorn the graphene layers, was determined using a LogNormal size distribution function [45], as depicted in Figure S1 in the Supplementary Materials, yielding a result of D = 22.23 nm.

**Figure 2:**
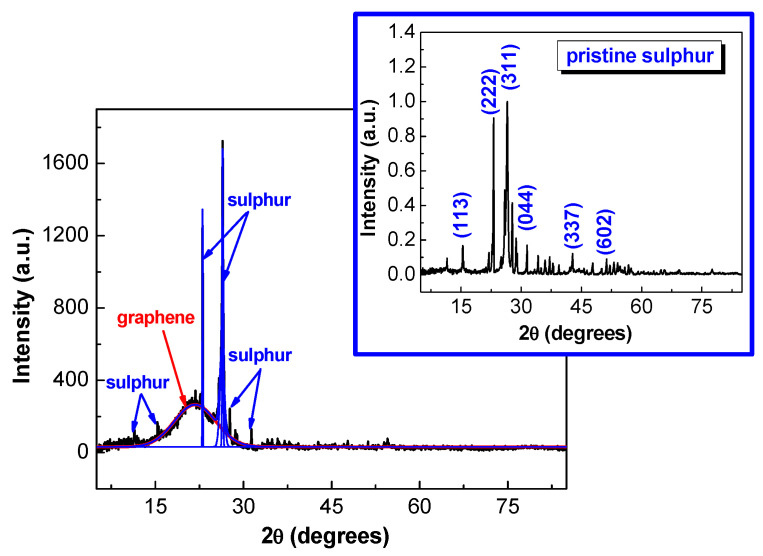
XRD pattern corresponding to exfoliated sulphur-doped graphene sample with the deconvolution of the most intense feature; inset: pristine sulphur XRD pattern (JCPDS no. 08-0247).

The diffractometric study (Figure 2) reveals the formation of few-layer graphenes (FLG)—since the XRD pattern is dominated by a broad diffraction peak at 21.74, corresponding to the reflections of (002) planes of graphene [46], along with the intense sulphur lines (at 11.55°, 15.46°, 23.16°, 26.46°, 27.76°, and 31.46°—JCPDS card no. 77-0145). Based on the deconvolution of the corresponding graphene peak (which appears between 5 and 35 degrees) and using the Debye–Scherrer equation [47], the mean crystallite size was determined to be 1.203 nm. Furthermore, the number of layers in the graphene sheets was found to be 2.94, with an interplanar distance calculated according to the Bragg equation [48] of 0.408 nm. This value is considerable higher compared to that reported for graphite (0.334 nm) [49], due to the defects induced by sulphur crystallites and oxygen-containing groups.

**Figure 3:**
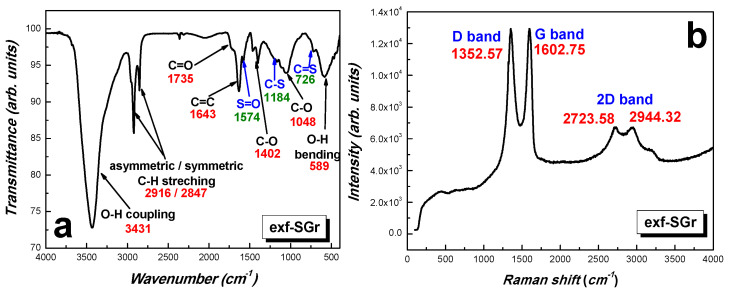
FTIR spectrum (**a**) and Raman band identification (**b**) in exfoliated sulphur-doped graphene.

The FTIR spectrum of sulphur-doped graphene (see Figure 3a) is dominated by a major absorption peak located at 3431 cm^−1^ and assigned to the O-H coupling. Two other intense spectral features centered at 2916 cm^−1^ and 2847 cm^−1^ correspond to the asymmetric/symmetric stretching vibration of C-H bonds in –CH_2_ groups, while the absorption peak at 1643 cm^−1^ represents the out-plane vibration of the –CH_2_ group and the asymmetric deformation vibration. Absorption peak characteristics for C=S, C-S, and S=O vibrations were observed at 726 cm^−1^, 1184 cm^−1^, and 1574 cm^−1^, respectively, confirming the sulphur doping of the graphene lattice.

Sulphur-doped graphene material displays four characteristic peaks on its Raman spectrum profile (see Figure 3b), typical for few-layers graphene, with defects [50], namely, the D band at 1352 cm^−1^—a measure of disorder and structural defects; the G band at 1602 cm^−1^—a quality parameter for the amount of graphene layers; and the 2D-band doublet located at 2723 cm^−1^ and 2944 cm^−1^. The intensity ratio of the D and G bands (I_D_/I_G_ = 1.010) is significantly higher compared to other values reported in the literature [51,52], reflecting the considerable number of defects induced by the high doping degree, which breaks the ideal bi-dimensionality of a graphene structure. In addition, some Raman band shifts appeared compared to other reports in the literature [53] due to defects and disorder induced by p-type doping in the graphene structure. According to the Tuinstra and Koenig relation [54], the I_D_/I_G_ ratio is inversely proportional to the crystallite size La: I_D_/I_G_ = C/L_a_, where C is a constant that depends on the laser excitation energy, E_i_ (2.41 eV). In our case, the in-plane crystallite size was determined to be L_a_ = 16.78 nm, I_G_/I_D_ = 0.99, and I_G_/I_2D_ = 1.89. According to the Raman results, the few-layer graphene structure with a high crystallinity of the exf-SGR material is confirmed. Furthermore, the high intensity of the D band clearly indicates the presence of a high number of structural defects.

**Figure 4:**
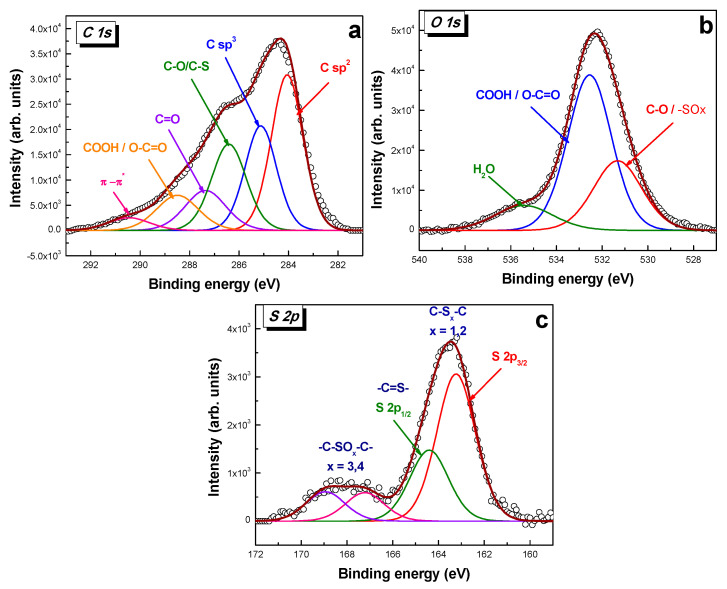
Deconvolution of the high-resolution C 1 s (**a**), O 1 s (**b**), and S 2p (**c**) XPS signals corresponding to the sulphur-doped graphene sample.

To confirm the presence of sulphur doping in the graphene lattice and provide insights into the structure of the graphene and the interaction between the sulphur atoms and the graphene lattice, XPS characterization was performed (see Figure 4). The high-resolution C1s spectrum (Figure 4a) was deconvoluted using six different Gauss–Lorentz components located at 284.05 eV (33.08%), 285.12 eV (22.23%), 286.41 eV (20.22%), 287.35 eV (11.01%), 288.49 eV (9.93%), and 280.27 eV (3.53%), accounting for C=C, C-C, C-O/C-S, C=O, O=C-O/COOH, and the π→π* transition, respectively [55,56]. As is visible, the most intense contribution in the carbon spectra appears at 284.05 eV, confirming the importance of sp^2^ hybridization in our material. The deconvolution of the O 1s spectrum (Figure 4b) reveals the presence of structural defects, which appear in the form of C-O, C=O, O-C=O, and (-SO_x_) bonding and are represented by the two different fit components located at 531.32 eV (29.03%) and 532.54 eV (58.54%). The adsorbed water molecules contribute 12.46% to the spectrum, shown as a peak in the high-binding-energy region, at 535.42 eV. According to the S 2p spectrum deconvolution (Figure 4c), the sp^2^ carbon lattices were successfully modified to incorporate S-containing functionalities. The two major spectral features contain contributions from both carbon–sulphur bounding (C-S_x_-C, where x = 1, 2; C=S) and oxidized sulphur moieties (-SO_x_, where x = 3, 4) [57,58]. The deconvolution was made according to the spin-orbit splitting with a doublet of lines to accommodate for S 2p_3/2_ (163.23 eV—53.32%) and S 2p_1/2_ (164.41 eV—25.74%), respectively [59]. Furthermore, the high-binding-energy component was also fitted with two peaks centered at 167.22 eV (10.31%) and 168.93 (10.63%), corresponding to oxidized sulphur species [60]. The C/O ratio was determined to be 1.05, while the C/S was 11.57, confirming a significant number of defects in the graphene lattice and the high heteroatom doping degree, respectively.

### 2.2. Development of Electrochemical Detection Protocol for Ferulic Acid Assay on the Surface of Modified Electrode

#### 2.2.1. Graphene-Modified Electrode Surface Characterization

An important role in understanding the electrocatalytic activity of a modified electrode surface requires determining its active area. As presented in Figure S2a from the Supplementary Materials, cyclic voltammetry measurements were carried out for this purpose at various scan rates, ranging from 2 to 100 mV/s in 0.2 M KCl of supporting electrolyte, containing 1 mM of K_4_[Fe(CN)_6_] as the redox indicator. A linear dependence, described according to the following regression equation, Ipa = 1.57 × 10^−6^ + 3.05 × 10^−5^ × υ^1/2^ (R^2^ = 0.996), was observed in the case of the anodic peak current versus the scan rates’ square root—see Figure S2b; and the active area was determined based on the Randles–Ševčík equation [61] to be: 0.0457 cm^2^; a considerably higher value was reported for the unmodified electrode (0.028 cm^2^). Comparing the active areas obtained in the case of several modified electrodes, it was observed that the differences do not exceed 7.37%, proving the good reproducibility of the electrode modification and the immobilization of the exfoliated sulphur-doped graphene material on the glassy carbon surface. 

In order to obtain information about the charge-transfer resistance (R_ct_) of the exf-SGR material deposited on top of the GCE, the EIS technique was employed. In Figure S3 from the Supplementary Materials, the EIS spectra (Nyquist plot) of the bare GCE (blue) and the exf-SGR-modified electrode (red) are comparatively presented. As can be observed, there are significant differences between these two plots. In the case of the bare GCE, the graph reveals a semi-circle in the high–medium frequency range, due to the large charge-transfer resistance of the electrode surface. At low frequencies, a different phenomenon occurs, which is the diffusion of the redox species within the double-layer, as described by the Warburg impedance (W). A constant phase element (CPE) was added to describe the charging of the double-layer formed near the electrode surface, and a resistor for the solution resistance (Rs) (Inset a of Figure S3 from the Supplementary Materials). In the case of the exf-SGR/GCE, the equivalent electrical circuit (see Inset b of Figure S3 from the Supplementary Materials) contains the same elements, but the Warburg impedance was replaced by another constant phase element (CPE_2_), which generated a better fit of the experimental data. The straight line observed for the exf-SGR/GCE, with low real and imaginary values, may be related to the fact that the transfer of electrons between the molecules in the diffusion layer and the electrode surface is very fast. This was confirmed by the *R_ct_* values obtained after fitting the EIS spectra with the equivalent electrical circuits: 36.5 kΩ (GCE) and 48.7 Ω (exf-SGR/GCE). After the *R_ct_* values for each electrode were determined, the corresponding apparent heterogeneous electron transfer rate (*k_app_*) was calculated using the following equation [62]:

$$k_{app}=\frac{RT}{n^{2}F^{2}AR_{ct}C}$$

where *n* is the number of electrons transferred during the redox reaction (*n* = 1); *F* is the Faraday constant (96,485 C mol^−1^); *R* is the ideal gas constant (8.314 J mol^−1^K^−1^); *T* is the temperature (298 K); *A* is the active area of the electrode (cm^2^); *R_ct_* is the charge-transfer resistance obtained from the fitted Nyquist plots (Ω); and *C* is the concentration of the redox species (mol·cm^−3^).

In the case of the GCE, *k_app_* was determined to be 2.6 × 10^−4^ cm/s, while for the exf-SGR/GCE, *k_app_* was 1.09 × 10^−1^ cm/s. One can see that the graphene-modified electrode has the value of *k_app_* three orders of magnitude higher than that corresponding to the bare GCE, clearly indicating that the deposited graphene flakes highly promote the transfer of electrons between the redox molecules and the glassy carbon substrate.

#### 2.2.2. Ferulic Acid Electrochemical Oxidation at the Surface of exf-SGR/GCE

The number of anodic oxidation peaks, their assignment, and the oxidation process of ferulic acid are all highly debated topics in the literature. Liu et al. [35] took into account a pair of redox peaks located at 481 mV (anodic) and 392 mV (cathodic), while a broad, higher potential anodic peak was ascribed to the adsorbed FA molecules. A pair of redox peaks, at 370 mV and 260 V, was also considered in the work of Li et al. [63]. In the voltammetric study of Trabelsi et al. [64], two oxidation peaks were visible at 240 and 270 mV, respectively, accompanied by a broad reduction peak visible at 150 mV. Manaia and collaborators [65] described two consecutive charge-transfer reactions at 510 mV and 650 mV in the oxidation pathway together with the corresponding reduction signals at 540 and 310 mV, suggesting a reversible redox reaction. A similar ferulic acid oxidation behavior, involving two consecutive oxidation steps (at 363 and 466 mV), was reported in the work of Tomac et al. [66]. The only common view in all studies is represented by the fact that FA molecules are electrochemically oxidized by electron transfer in both free and adsorbed forms. The first oxidation peak corresponds to the oxidation of FA molecules in their free form, whereas the second anodic peak corresponds to the oxidation of FA molecules in their adsorbed state.

**Figure 5:**
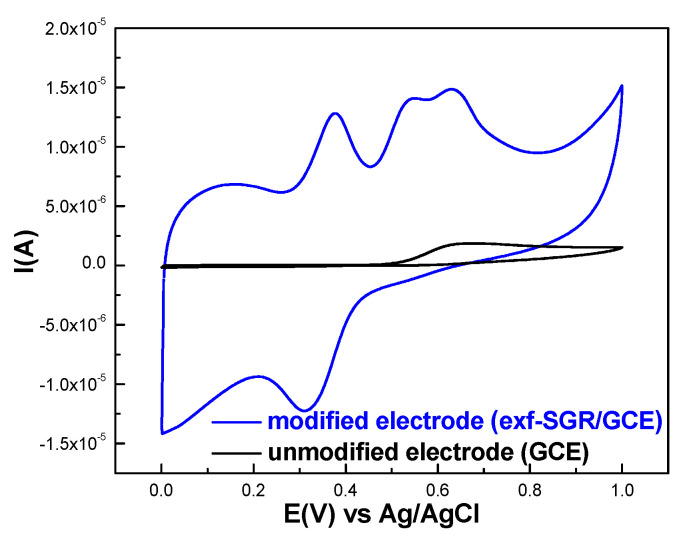
CVs corresponding to the bare glassy carbon electrode and exf-SGR-modified electrode in the presence of 100 µM FA concentration; scan rate of 10 mV/s.

In the case of the exf-SGR/GCE surface, a remarkable enhancement of the electrochemical response was observed compared to the bare electrode in the presence of 100 µM FA in pH 5 acetate buffer solution (see Figure 5). The unmodified surface generated a broad anodic response centered at about 662 mV, and no reduction was recorded. In contrast, the electrochemical behavior of the sulphur-doped graphene-based sensor is characterized by the presence of a sharp anodic response at 376 mV (assigned to the oxidation of FA molecules from the solution) and an overlapping of oxidation contributions with two visible maxima at 542 mV and 637 mV, accounting for the electro-oxidation of FA molecules adsorbed on the electrode surface [64].

**Figure 6:**
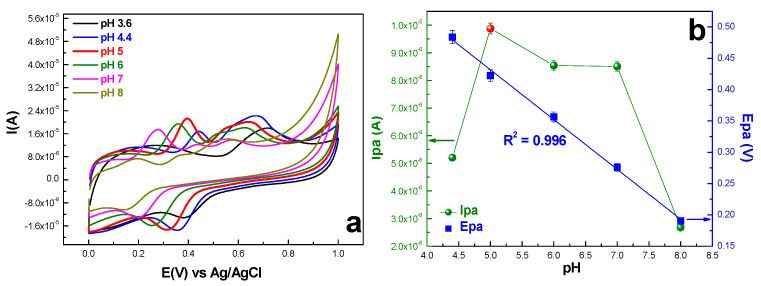
(**a**) CV response of exf-SGR/GCE surface, recorded in acetate (pH 3.6; 4.4; 5) and phosphate (6; 7; 8) buffer solutions containing 100 µM FA; scan rate 10 mV/s; (**b**) dependence of anodic peak current and potential versus pH value.

The pH effect on the FA electrochemical detection on the surface of sulphur-doped graphene-modified GCE was analyzed by CV in different buffer solutions (acetate and phosphate), with pH changing from 3.6 up to 8.0. As shown in Figure 6a, the presence of 100 µM FA in the working solution, generates, in all cases, well-defined anodic and cathodic peaks suggesting a quasi-reversible redox process on the surface of the S-doped graphene-based modified electrode. Similar electrochemical behavior was reported by Tomac et al. [66], Bounegru [37], and Da Silva et al. [67]. From our point of view, it was important to follow up the evolution of lower potential sharp oxidation feature, since, according to the literature [64], this corresponds to the electro-oxidation of FA species from the solution. Furthermore, it was previously stated that the electrochemical polymerization of FA at the electrode surface is an inevitable result of the electrochemical oxidation process [63]. FA had two pKa values 4.53 and 8.95 [68] and existed as a neutral molecule at pH levels ranging from 1 to 3. As is visible, in the case of pH 3, FA only generated a broad contribution located at 0.7 V and a small reduction peak. As the pH of the working solution increased, so did the corresponding anodic peak current intensity, up to a maximum recorded at pH 5; after which, it slowly decreased, as depicted in Figure 6b (green line). Thus, this was chosen as the optimal condition, and all the experiments were further performed in pH 5 acetate buffer. When the pH rose above 8, the CV anodic peak current vanished; since, at high pH values, FA converts to anions. Thus, due to the lack of protons, an electrostatic repulsion appeared between the analyte and the modified surface, causing FA desorption from the electrode. Similar results were obtained in the study of Zhang et al. [69]. Furthermore, the anodic peak current position shifted in a negative direction as the pH increases, confirming the involvement of protons in the electrochemical process, which occurs on the exf-SGR/GCE surface. As a result, the peak potential follows the linear regression equation: Epa = 0.829 − 0.079 × pH (R^2^ = 0.996)—see the blue line in Figure 6b. 

**Figure 7:**
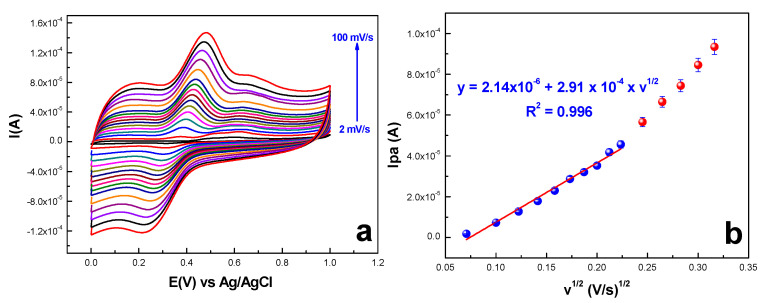
(**a**) Scan rate effect on the voltammetric response of exf-SGR/GCE surface toward 100 µM FA in pH 5 acetate buffer; (**b**) dependence of anodic peak current versus scan rate.

The scan rate influence on the oxidation current intensity was studied in the range of 2–100 mV/s, for 100 µM FA in pH 5 acetate buffer solution, as depicted in Figure 7a. As the scan rate increased, the intensity of the anodic and cathodic peaks’ current increased. Furthermore, it was very interesting to see that the scan rate influences the intensity of the anodic peak centered at about 372 mV, which significantly increases compared to the high-potential anodic features. The plot of the anodic peak current versus the square root of the scan rate obeys a linear relationship according to Ipa = 2.14 × 10^−6^ + 2.91 × 10^−4^ × υ^1/2^ (R^2^ = 0.996) in the scan range of 2–50 mV/s—see Figure 7b, which confirms a diffusion-controlled process [70]. When the scan rate exceeds 50 mV/s, a change in the process appears. Furthermore, a small shift of the anodic and cathodic signals toward the positive and negative potential, respectively, was recorded while increasing the scan rate. 

**Scheme 1:**
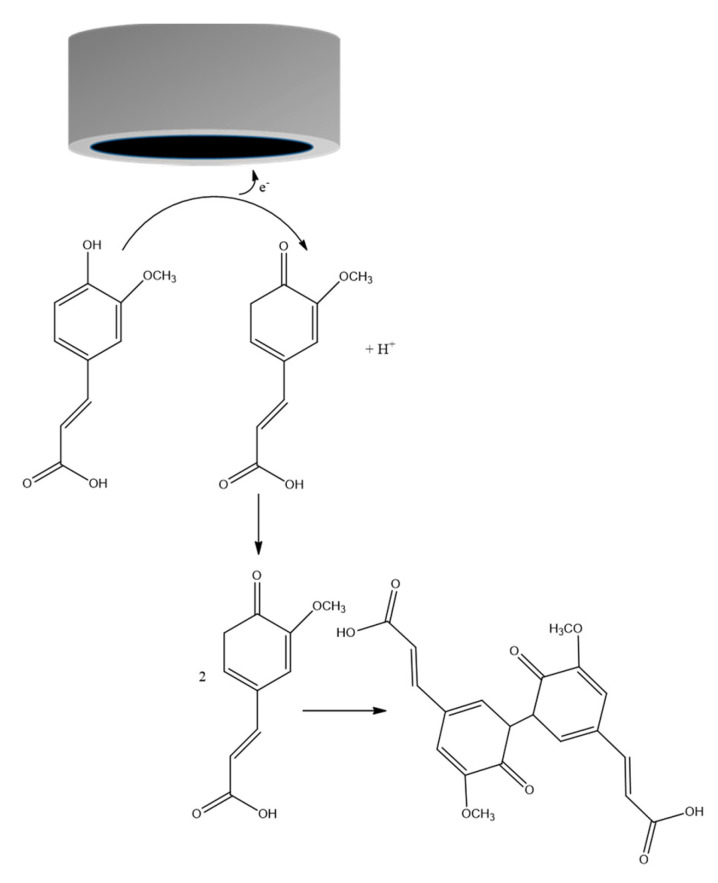
Electrochemical oxidation mechanism of FA at exf-SGR/GCE surface.

Based on all the data presented until now, a possible mechanism of the FA electrochemical oxidation at the surface of the exf-SGR/GCE sensor was proposed according to Scheme 1.

#### 2.2.3. Quantification and Detection Limits

**Figure 8:**
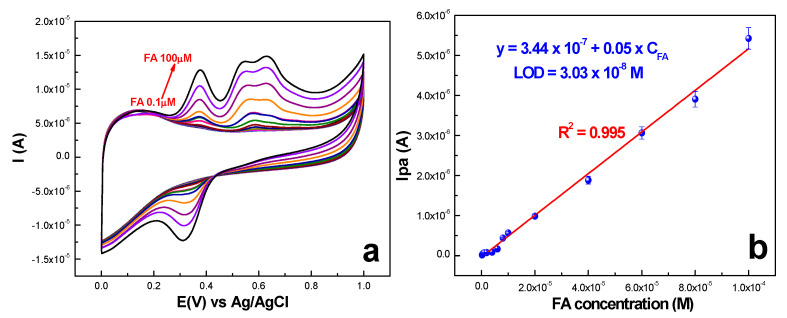
(**a**) CVs of the exf-SGR/GCE toward different FA concentrations—(0.1–100) µM—in acetate buffer pH 5; (**b**) the linear relationship between oxidation peak current and FA concentration.

Under the optimum experimental conditions, the voltametric technique was further employed to test the ability of the exf-SGR/GCE surface in detecting and quantifying ferulic acid at various concentrations in the range of 0.1–100 µM (see Figure 8a). As the FA concentration in the working solution increased, so did the voltammetric anodic signal centered at 376 mV and its corresponding reduction from 311 mV. The linear relationship between the anodic oxidation peak current and the concentration is described by Ipa = 3.44 × 10^−7^ + 0.05 × C_FA_ (R2 = 0.995)—Figure 8b. The limit of quantification (LOQ) was determined to be 0.1 µM, while the limit of detection (LOD) was 0.0303 µM. The obtained values are acceptable limits for the FA assay in real sample analysis. 

The electrochemical capabilities of the developed sulphur-doped graphene-based sensor were compared with the previous results reported in the literature (see Table 1). As is visible, the developed sensor exhibits comparable or even superior performances not only in terms of the linear range but also the limit of detection.

#### 2.2.4. Stability, Reproducibility, and Interference

The reproducibility of the graphene-modified surface was tested evaluating the electrochemical response of five different electrodes, obtained in the same conditions, toward 100 µM FA (see Figure S4a from the Supplementary Materials). As is visible, the differences in the obtained electrochemical signal did not exceed 4.72%, confirming the good reproducibility of the surface modification process. Furthermore, in order to evaluate the intra-day stability and reproducibility of the exf-SGR/GCE surface, the same electrode was subjected to several CV measurements in pH 5 acetate buffer solution containing 100 µM FA (see Figure S4b from the Supplementary Materials). Between measurements, the electrode was desorbed by cycling in pH 5 acetate buffer solution. It was observed that the developed sensor shows a very good intra-day stability and that the differences in the recorded electrochemical signal are not significant. In addition, the stability was also tested by subjecting the same electrode to 50 consecutive scan cycles at a scan rate of 10 mV/s. The result can be seen in Figure S4c from the Supplementary Materials. The exf-SGR/GCE surface retained 96.58% of its initial response, again proving its excellent time stability. The inter-day performances of the developed sensor were evaluated by performing the same measurements, in replicated experimental conditions, from time to time, for a longer time period of more than five months (Figure S4d from the Supplementary Materials). Again, it was proved that the developed modified electrode provides a stable response toward FA, since 97.28% of its initial response was preserved after more than 5 months.

It was critical to assess a sensor’s utility by testing its selectivity in the presence of possible interfering species. In the case of the exf-SGR/GCE-modified electrode, the effect of both organic (glucose; uric acid; citric acid; tartaric acid; caffeic acid) and inorganic (Na_2_CO_3_; NaCl; MgSO_4_; MgCl_2_; Mg(NO_3_)_2_) interferences at a given concentration of 100 µM, on the detection of a similar amount of ferulic acid, was evaluated. As shown in Figure S5 from the Supplementary Materials, the majority of the investigated interfering species generate no electrochemical signal at the surface of developed sensor, except for uric (Figure S5g) and citric acids (Figure S5h), which determine the apparition of a very small peak located at about 471 and 476 mV, respectively. Furthermore, the modified electrode was able to detect FA without being affected by the presence of common interfering species, confirming the good selectivity of the sulphur-doped graphene surface toward ferulic acid (see Figure S6 from the Supplementary Materials).

#### 2.2.5. Real Sample Analysis

**Figure 9:**
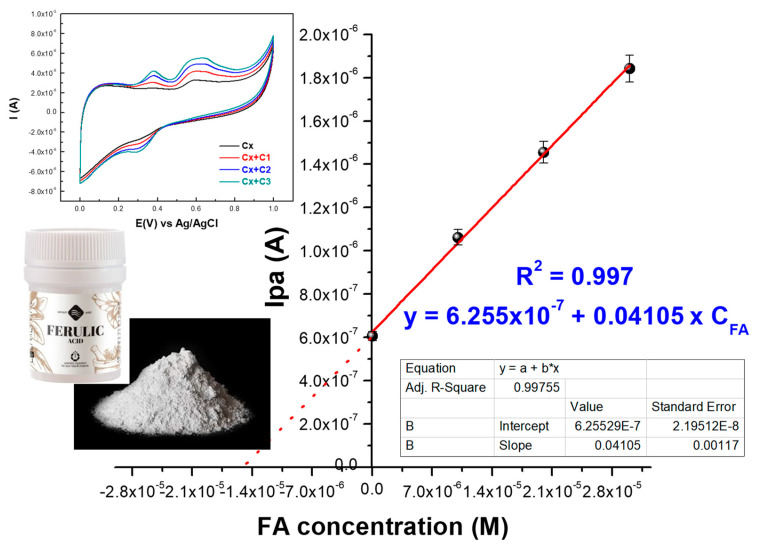
Standard addition method applied to real sample obtained from commercially available pharmaceutical ferulic acid formulation. Inset: cyclic voltammograms obtained in real sample solution spiked with different amounts from a stock ferulic acid solution (1 mM)—scan rate of 10 mV/s; supporting electrolyte pH 5 acetate buffer.

The practical applicability of the developed graphene-based sensing platform was tested in real sample analysis. A commercially available FA pharmaceutical formulation was employed in order to obtain a real sample solution of −0.1 mg/mL FA. The concentration of the as-prepared sample was calculated to be 0.515 mM. Next, aliquots (100 µL) of the as-prepared solution were transferred to four different beakers. The first beaker contained only the real sample regarded as the unknown concentration that needed to be determined (C_x_ = 1.03 × 10^−5^ M), while the rest of the beakers were spiked with known amounts (50, 100, and 150 µL) of FA from a stock solution of 1 mM. Further, all beakers were filled with pH 5 acetate buffer solution up to a final volume of 5 mL. Afterwards, CV measurements were recorded on the as-prepared solutions, and the results are shown in the inset of Figure 9. The unknown concentration of the real sample solution was determined by plotting the oxidation peak current versus the FA concentration, resulting in 1.52 × 10^−5^ M. In the case of cyclic voltammetry experiments, the residual standard deviation was determined to be 0.889.

Further, the validity of electrochemical results was compared with the one obtained using a complementary analytical technique, namely, UV–Vis spectroscopy (see Figure S7 from the Supplementary Materials). In the case of spectroscopic experiments, based on the work of Goujot et al. [77], the DPPH^•^ test was employed. First, a calibration curved was obtained by quantifying the reduction of DPPH^•^ at different known FA concentrations (ranging between 5–50 µM), and, based on this, the concentration of FA in the real sample solution was determined to be 1.15 × 10^−5^ M, while a residual standard deviation of 0.364 was calculated.

All the obtained results suggest that this research has yielded a cost-effective and sensitive electrochemical tool capable of determining with a high accuracy, rapidity, and selectivity low levels of FA, potentially offering a promising solution for the FA assay in the pharmaceutical and food sectors and biological sample analysis.

## 3. Materials and Methods

### 3.1. Reagents

Graphite rods 6 mm in diameter (99.995%); sodium thiosulfate (Na_2_S_2_O_3_, ≥99.0%) potassium chloride (KCl, 99.98%); trans-ferulic acid (FA; C_10_H_10_O_4_; 99%); uric acid (C_5_H_4_N_4_O_3_, ≥99%), caffeic acid (C_9_H_8_O_4_, ≥98.0% HPLC); 2,2-diphenyl-1-picrylhydrazyl (DPPH, C_18_H_12_N_5_O_6_, 99.98%); and L-(+)-Tartaric acid (C_4_H_6_O_6_, ≥99.5%) were purchased from Sigma-Aldrich (Taufkirchen, Germany). Sodium dihydrogen phosphate (H_2_NaO_4_P, 100%) and di-sodium hydrogen phosphate anhydrous (HNa_2_O_4_P, 99.7%) were bought from VWR Chemicals (Leicestershire, Belgium). Sodium carbonate (Na_2_CO_3_; 99.98%); sodium chloride (NaCl; 99.99%); sodium acetate anhydrous (CH_3_COONa, ≥99.0%); sodium thiosulfate pentahydrate (Na_2_S_2_O_3_·5H_2_O; 99%); magnesium sulphate (MgSO_4_; 99%); magnesium dichloride (MgCl_2_; 99.98%); magnesium nitrate (Mg(NO_3_)_2_; 99.99%); and ammonium sulphate ((NH_4_)_2_SO_4_, ≥99.0%) were provided by REACTIVUL Bucuresti (Bucharest, Romania). Citric acid (C_6_H_8_O_7_, 99+%); methanol HPLC grade (CH_3_OH); and uric acid (C_5_H4N_4_O_3_, 99%) were purchased from Alfa-Aesar (Karlsruhe, Germany), while potassium hexacyanoferrate (III) (K_4_[Fe(CN)_6_] ≥ 99%), L(+)-Ascorbic acid (C_6_H_8_O_6_, ≥99%), and D-(+)-glucose (C_6_H_12_O_6_; 99.5%) were acquired from Merck (Darmstadt, Germany). In order to prepare the working solutions, the reagents were used without any purification in ultrapure water (18.2 MΩ × cm at 25 °C), obtained from a Milli-Q system (Millipore Corp., Bedford, MA, USA).

### 3.2. Sulphur-Doped Graphene Preparation

In order to obtain highly doped sulphur few-layer graphene sheets, an electrochemical protocol was developed based on the exfoliation of high-purity graphite rods at low applied bias (5 V) in mixture of ammonium sulphate/sodium thiosulfate ((NH_4_)_2_SO_4_/Na_2_S_2_O_3_ (0.3 M each). Shortly, 3.96 g (NH_4_)_2_SO_4_ and 7.44 g Na_2_S_2_O_3_ were dissolved in a volume of 100 mL ultrapure water. Next, two graphite rods were fixed at a distance of 2 cm with a homemade Teflon cap in a 150 mL beaker filled with the previously prepared solution and coupled to a Mesit (Uherské Hradiště, Slowakia) power source. For the next 3 h, a DC voltage (5 V) was applied to the electrolytic cell. Shortly after the beginning of the experiment, the exfoliation process of graphite started, and a dark-colored material appeared at the bottom of the electrochemical cell. Since the quantity of the exfoliated material increased, the electrolyte changed color from clear to black solution. After 3 h, the experiment was stopped, the remaining graphite rods were removed from the cell, and the obtained solution was allowed to rest overnight at room temperature. The next day, since the two phases separated, we removed the supernatant, and the exfoliated material remained at the bottom of the electrochemical cell. Following, ultrapure water washing procedure was applied until a neutral pH was reached. In the next step, the washed material was dispersed in 100 mL of ultrapure water, sonicated for 30 min, and filtered on red ribbon Whatman qualitative filter paper (589^5^). The filtered solution was sonicated for 15 min and evaporated under magnetic stirring, at 50 °C, until the final volume was reduced to about 30 mL. The resulting solution was frozen and dried by lyophilization, until a black powder sample was obtained (denoted exf-SGR). 

### 3.3. Sensor Design

For the obtaining of graphene-based modified sensitive surfaces, glassy carbon electrodes (GCE) were polished on felt cloth, rinsed with ultrapure water, and dried at room temperature. Afterwards, sulphur-doped graphene suspension prepared in DMF (1 mg/mL) was deposited by drop-casting method on top of working glassy carbon electrode. Shortly, batches of 0.5 µL were successively pipetted and dried at room temperature until a total volume of deposit solution of 8.5 µL was reached. After 24 h, the modified electrode denoted exf-SGR/GCE was ready to be used in electrochemical detection experiments. Between different measurements, the modified electrode surface was cleaned by cycling in pH 5 buffer solution, and the electrode was continuously kept in ultrapure water when not in use. The reproducibility of electrode surface modification was checked by preparing several electrodes in the same conditions and testing their active surface areas and electrochemical performances. Furthermore, optimization of sulphur-doped graphene’s total volume deposited on glassy carbon electrode surface was performed in the range of 5–15 µL. It was observed that volumes smaller than 6 µL are not enough to efficiently cover up the entire surface, while for volumes higher than 8.5 µL the heavy covering impedes the redox process.

### 3.4. Apparatus

The sample produced following electrochemical exfoliation of graphite rods was dried using a Christ-Alpha 1-4 LSC freeze-drier (Martin Christ Gefriertrocknungsanlagen GmbH, Osterode am Harz, Germany). The sulphur-doped exfoliated-graphene morphology was revealed by Transmission Electron Microscopy (TEM) and Scanning Electron Microscopy (SEM), while Scanning Transmission Electron Microscopy (STEM) with Energy Dispersive X-ray (EDS) Spectroscopy technique was employed for the elemental mapping of C, O, and S atom distribution. The measurements were performed using an HD 2700 Hitachi system (Hitachi High-Technologies Corp., Tokyo, Japan). A Brucker D8 Advance Diffractometer (40 kV; 0.5 mA) outfitted with a LYNXEYE detector (λ = 1.5406 Å) provided the X-ray diffraction (XRD) data. X-ray Photoelectron Spectroscopy (XPS) experiments were conducted on a SPECS spectrometer outfitted with a dual-anode X-ray source Al/Mg, a PHOIBOS 150 2DCCD hemispherical energy analyzer, and a multi-channeltron detector, while being irradiated with an AlK X-ray source (1486.6 eV) operating at 200 W. No smoothing of the data was performed, and the binding energies are provided without correction, since no electrostatic charge appeared on the sample surface during measurement. Casa XPS software—version 2.3.16 (Casa Software Ltd., Wilmslow, Cheshire, UK) was used for data analysis and experimental curve fitting of the C 1s and O1s spectra. A Gaussian–Lorentzian profile and a non-linear Shirley background correction were considered. SPECORD 250 PLUS spectrophotometer (Analytik Jena GmbH, Jena, Germany), in the 200–800 nm spectral range was employed for the UV–Vis analysis. Electrochemical testing was performed with a Potentiostat/Galvanostat PGSTAT-302N (Metrohm-Autolab B.V., Utrecht, The Netherlands), using a typical three-electrode cell, whereas Ag/AgCl electrode was set as reference; a large platinum sheet was employed as counter electrode, and the bare or graphene-modified glassy carbon electrode (3 mm diameter) served as the working electrode.

## 4. Conclusions

An electrochemical detection protocol for the rapid, highly selective, and sensitive assay of ferulic acid was developed based on the usage of a novel sulphur-doped graphene sensor. The electrode material preparation consisted of the electrochemical exfoliation of graphite, at a low applied bias (5 V) in the presence of ammonium sulphate/sodium thiosulfate (0.3 M each). According to the morphological characterization and XRD analysis, the obtained material appeared as a highly sulphur-doped few-layer graphene. The Raman spectroscopy results confirmed the existence of a considerable number of defects in the 2D graphene lattice. The high doping degree and the existence of defects were further confirmed by means of the XPS deconvolution of the C1s, O1s, and S2p levels. The C/O and C/S ratios were determined to be 1.05 and 11.57, respectively. Further, the analytical applicability of the as-prepared sensor was tested both in laboratory solutions and by real pharmaceutical sample analysis. Under optimized conditions, the exf-SGR/GCE-modified electrode showed a low limit of detection, 30.3 nM, in a wide linear range (0.1–100 µM) and good selectivity and reproducibility, demonstrating that it is a viable option for rapid FA detection, with applications in the food industry, as well as the pharmaceutical and health sectors.

## Figures and Tables

**Table 1:** Performance comparison of proposed exf-SGR/GCE sensor with other sensors in the literature.

**Electrode**	**Linear Range** **(µM)**	**Detection Limit** **(µM)**	**Technique**	**Reference**
**ERGO/GCE** ERGO—electrochemically reduced graphene oxide GCE—glassy carbon electrode	0.08–38.9	0.02	DPV	[35]
**PDDA-G/GCE** PDDA-G—poly(diallyldimethylammoniumchloride) functionalized graphene GCE—glassy carbon electrode	0.09–52.5	0.02	DPV	[32]
**PAA/GCE** PAA—poly-aspartic acid GCE—glassy carbon electrode	0.91–3000	0.3	DPV	[33]
**TiO_2_/C/Au/GCE** TiO_2_/C/Au—Au-doped nanostructured TiO_2_/C material GCE—glassy carbon electrode	0–20.0	0.016	DPV	[71]
**rGO-TiO_2_/GCE** rGO-TiO_2_-TiO_2_ nanoparticles-decorated, chemically reduced graphene oxide	0.1–1.0	0.01	DPV	[72]
**rGO-AuNPs-MIP/SPE** rGO-AuNPs-MIP—molecularly imprinted polymer on reduced graphene oxide–gold nanoparticles SPE—screen-printed electrode	0.01–1.0	0.031	DPV	[73]
**MBIBr/NiO-SWCNTs/CPE** MBIBr/NiO-SWCNTs—NiO-embedded single-wall carbon nanotube nanocomposite and *n*-methyl-3-butylimidazolium bromide CPE—carbon paste electrode	0.06–900.0	0.02	SWV	[74]
**PPy-MWCNTs/GCE** PPy-MWCNTs—polypyrrole multi-walled carbon nanotube GCE—glassy carbon electrode	3.32–25.9	1.17	SWV	[34]
**MWCNT/GCE** MWCNT—multi-walled carbon nanotube	10–5000	1.17	CV	[75]
**MWCNTs-Ag/CPE** MWCNTs-Ag—multi-walled carbon nanotube decorated with silver nanoparticle-modified CPE—carbon paste electrodes	0.04–1000	0.03	CV	[69]
**DDAB/nafion/CPE** DDAB/nafion—Didodecyldimethyl-ammonium Bromide/Nafion Composite Film CPE—carbon paste electrode	2–1200	0.39	CV	[76]
**GOs/MWCNTs/GCE** GOs/MWCNTs—graphene oxide sheets (GOs) and multi-walled carbon nanotubes (MWCNTs) nanocomposite GCE—glassy carbon electrode	0.24–32 88–1230	0.08	CV	[31]
**GN/GCE** GN—graphene nanosheet GCE—glassy carbon electrode	0.5–50	0.2	CV	[68]
exf-SGR/GCE	0.1–100	0.0303	CV	current work

## Data Availability

The data presented in this study are available upon request from the corresponding author.

## Supplementary Materials

The following supporting information can be downloaded at https://www.mdpi.com/article/10.3390/ijms242316937/s1.
