# Supplementary material for: Graphene-Based Electrochemical Sensing Platform for Rapid and Selective Ferulic Acid Quantification
Source: Int J Mol Sci. 2023 Nov 29;24(23):16937. doi: 10.3390/ijms242316937 (PMC10707139; doi:10.3390/ijms242316937)
Supplement: Supplementary file 1 [file ijms-24-16937-s001.zip › ijms-2739911-supplementary.pdf]

Electronic Supplementary Materials

Graphene-Based Electrochemical Sensing Platform for Rapid and Selective Ferulic Acid Quantification

Lidia Măgeruşan *, Florina Pogăcean, Maria-Loredana Soran and Stela-Maria Pruneanu

National Institute for Research and Development of Isotopic and Molecular Technologies, Donat Street, No. 67-103, 400293 Cluj-Napoca, Romania
Correspondence: lidia.magerusan@itim-cj.ro

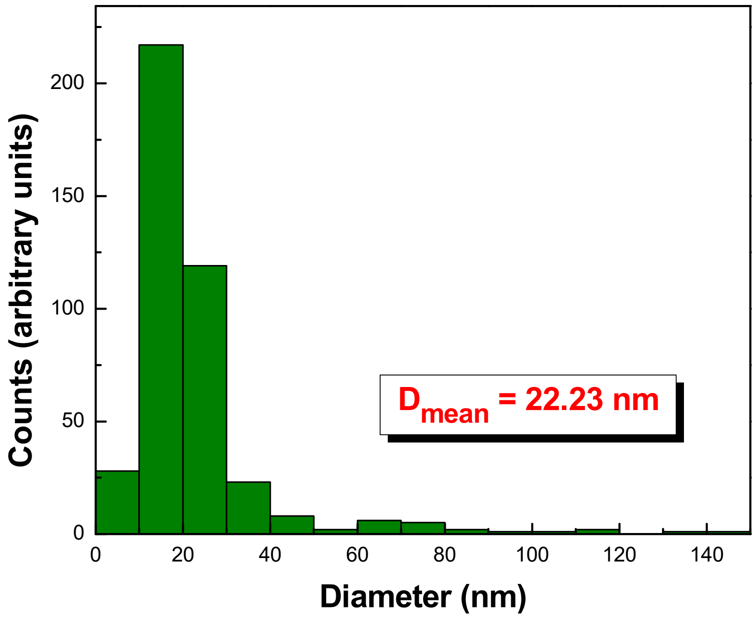

Figure S1. Size diameters histogram of sulphur nanoparticles.

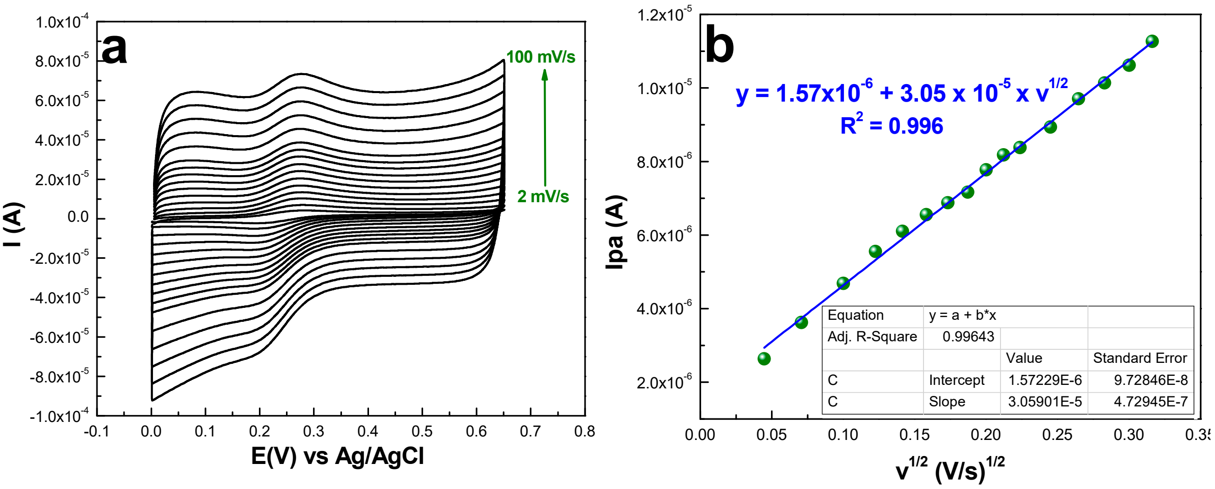

Figure S2. (**a**) Cyclic voltammetric response of exf-SGR/GCE surface towards 1 mM $K_4[Fe(CN)_6]$ in 0.2 M KCl supporting electrolyte, at various scan rates; (**b**) Ipa versus $\upsilon^{1/2}$ linear plot.

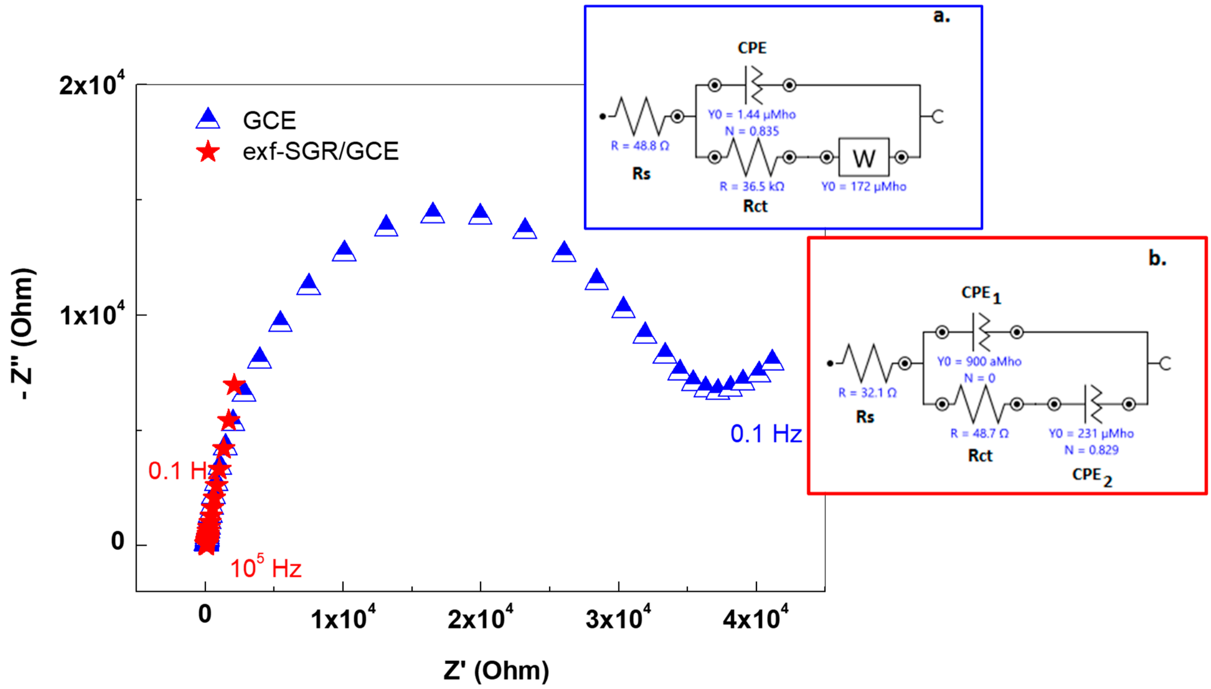

Figure S3. Nyquist plots obtained after recording the EIS data with bare GCE (blue) and exf-SGR/GCE (red), in solution containing 1 mM potassium ferrocynide and 0.2 M KCl. *Inset:* The equivalent electrical circuit employed to fit the bare GCE (**a**); the equivalent electrical circuit employed to fit exf-SGR/GCE (**b**).

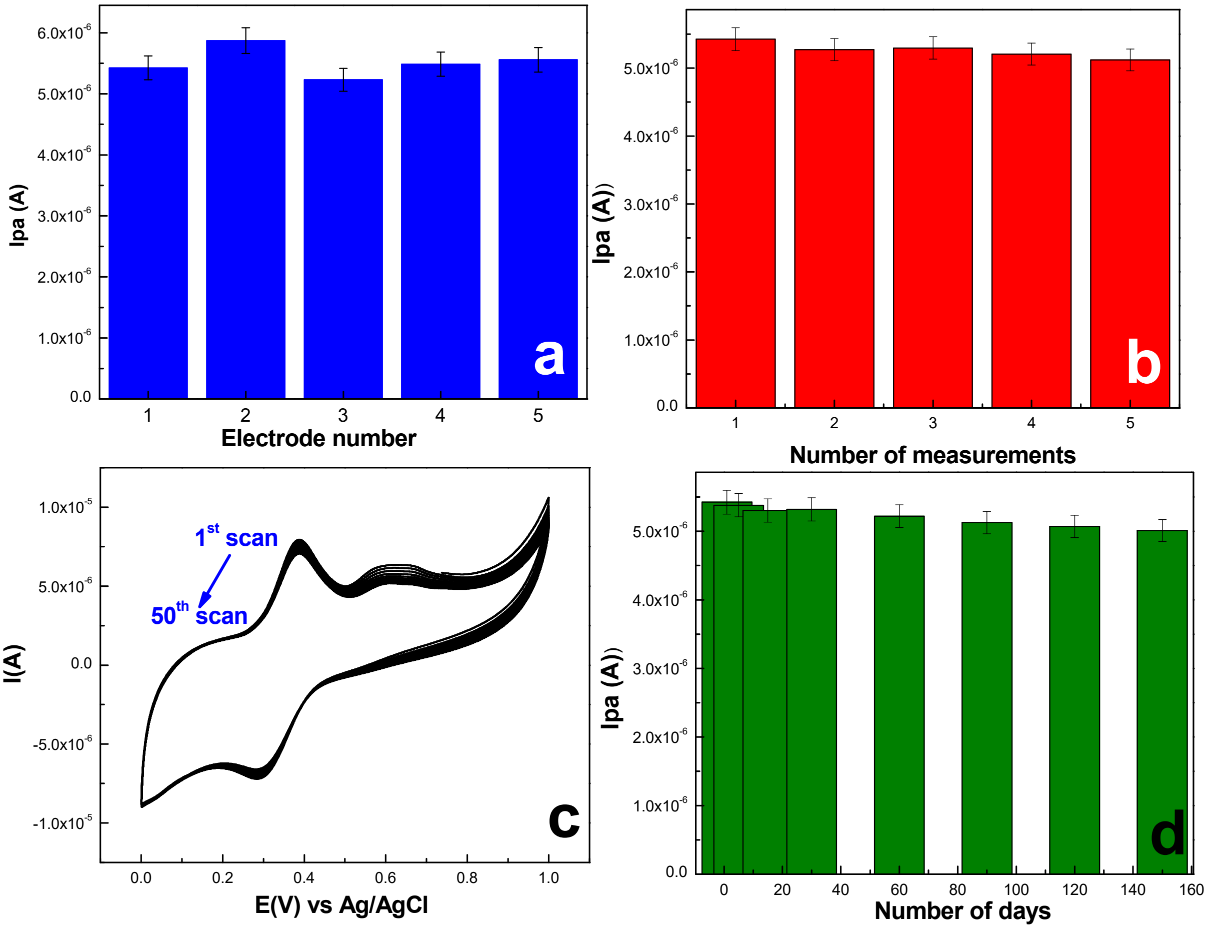

Figure S4. (**a**) Anodic peak current intensity obtained with five different electrodes; (**b**) Anodic peak current intensity recorded in five different measurements performed using the same electrode; (**c**) 50 consecutive cyclic voltammetry measurements recorded using exf-SGR/GCE modified electrode; (**d**) Cyclic voltametric response of exf-SGR/GCE obtained in replicate experiments over a long time period supporting electrolite: pH 5 acetate buffer solution containing 100 μM FA; scan rate 10 mV/s.

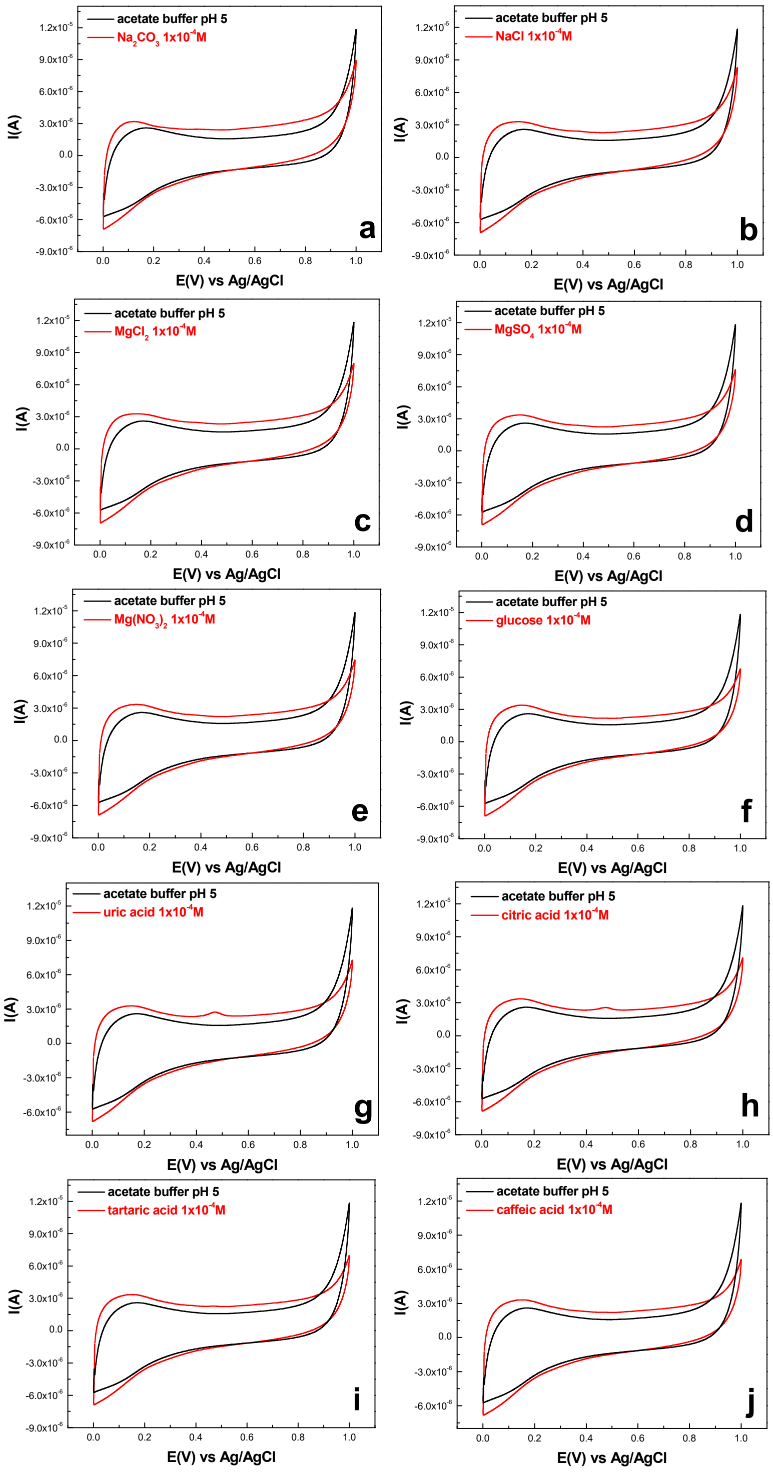

Figure S5. Electrochemical response of exf-SGR/GCE surface towards 100 μM (**a**) Na_2CO_3; (**b**) NaCl; (**c**) $MgCl_2$; (**d**) $MgSO_4$; (**e**) $Mg(NO_3)_2$; (**f**) glucose; (**g**) uric acid; (**h**) citric acid; (**i**) tartaric acid; (**j**) caffeic acid.

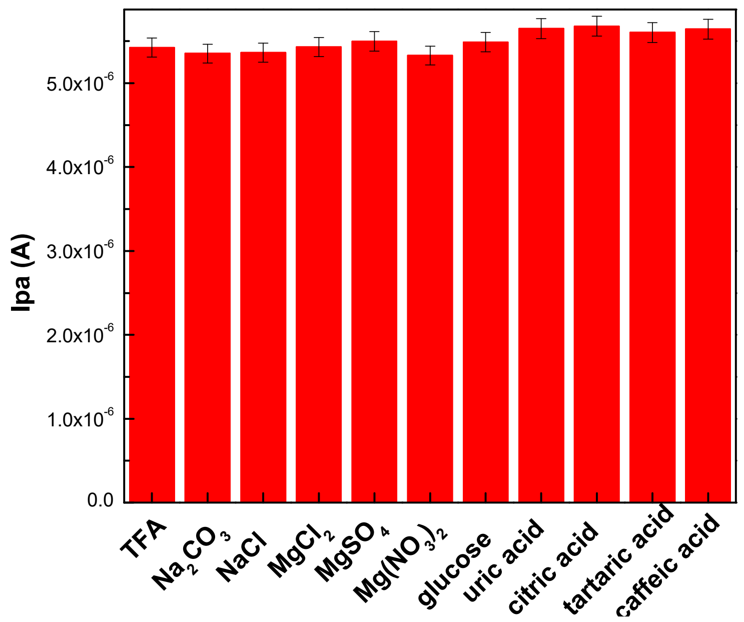

Figure S6. Annodic peak current intensity obtained towards 100 μM FA in pH 5 acetate buffer solution in presence of different organic and innorganic interfering species at a given concentration of 100 μM.

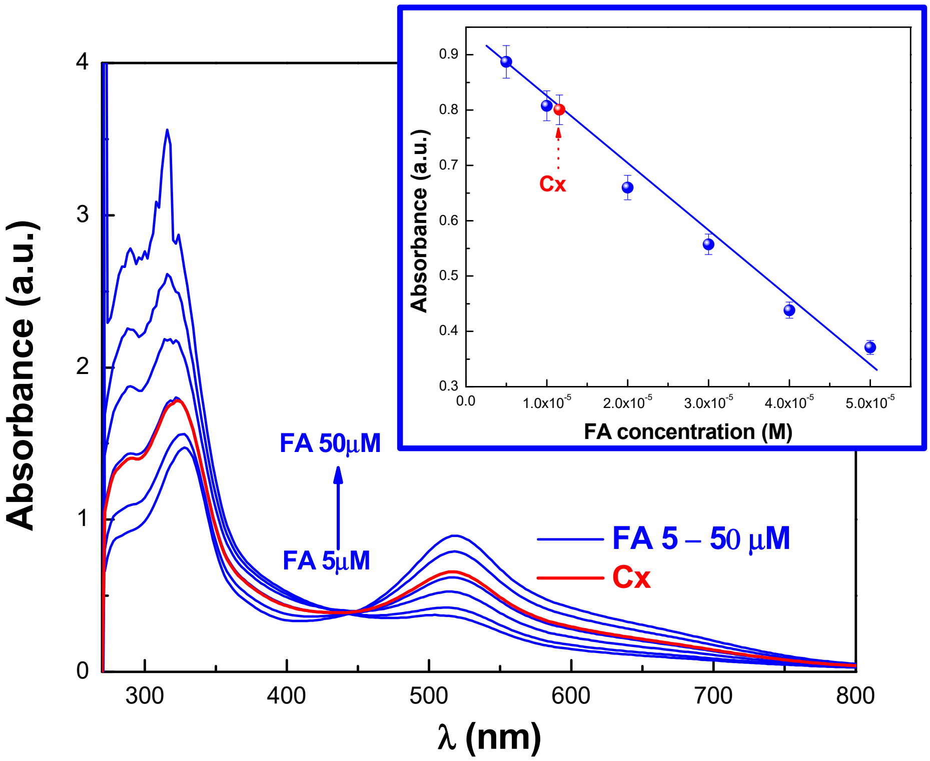

Figure S7. UV-Vis spectra recorded for known FA concentrations (5; 10; 20; 30; 40 and 50 μM) and for real sample obtained from commercially available pharmaceutical FA formulation. *Inset:* Corresponding calibration curve.
